# Immunohistochemical Analysis of Adhesion Molecules E-Selectin, Intercellular Adhesion Molecule-1, and Vascular Cell Adhesion Molecule-1 in Inflammatory Lesions of Atopic Dermatitis

**DOI:** 10.3390/life13040933

**Published:** 2023-04-02

**Authors:** Sandra Marinović Kulišić, Marta Takahashi, Marta Himelreich Perić, Vedrana Mužić Radović, Ružica Jurakić Tončić

**Affiliations:** 1 Department of Dermatology and Venereology, University Hospital Centre Zagreb, 10000 Zagreb, Croatia; 2 Department of Communicology, Catholic University of Croatia, 10000 Zagreb, Croatia; 3 Health Center Zagreb West, 10000 Zagreb, Croatia; marta.himelreich-peric@dzz-zapad.hr; 4 Hospital for Medical Rehabilitation of the Health and Lung Diseases and Rheumatism “Thalassotherapia-Opatija”, 51410 Opatija, Croatia

**Keywords:** atopic dermatitis, E-selectin, ICAM-1, VCAM-1

## Abstract

E-selectin, ICAM-1 (intercellular adhesion molecule-1), and VCAM-1 (vascular cell adhesion molecule-1) play a role in atopic dermatitis (AD). This study aimed to evaluate their expression in skin biopsy specimens of patients diagnosed with AD using an optimized computer program. A descriptive analysis and comparison of digitally measured surface area and cell number were performed. The number of E-selectin-positive cells did not vary between the groups. In patients with AD, decreases of 1.2-fold for ICAM-1- and 1.3-fold for VCAM-1- positive cells were observed. The E-selectin-positive epidermal surface area increased (*p* < 0.001), while ICAM1 and VCAM1 decreased 2.5-fold and 2-fold, respectively, compared to controls. In the AD-affected skin, the E-selectin-positive endothelial area was 3.5-fold larger (*p* < 0.001), and the ICAM1-positive area was almost 4-fold larger (*p* < 0.001). E-selectin and ICAM-1 were expressed in the control dermis moderately and weakly, respectively. A strong E-selectin signal was detected in the AD-affected skin macrophages and a strong ICAM-1 signal in the dermal vessel endothelium. In the endothelial cells of AD-affected skin, no VCAM-1 signal could be found. E-selectin, ICAM-1, and VCAM-1 expression show significant disease-specific changes between AD-affected and control skin. The combination of digital analysis and a pathologist’s evaluation may present a valuable follow-up of AD activity parameters.

## 1. Introduction

Atopic dermatitis (AD) is a chronic inflammatory disease mediated by type I and IV immunologic mechanisms in its pathogenesis [1]. A wide variation in the prevalence of AD is present in different populations of the world [2] but overall, it appears to be increasing [3,4,5]. AD is a skin disease with significant morbidity and quality-of-life impairment [6,7] and represents a healthcare burden. In 1980, Hanifin and Rajka proposed major and minor diagnostic criteria for AD [8] based on the history and clinical picture, determining the basis of the diagnostic criteria worldwide which has been re-evaluated since then [9,10,11].

The etiopathogenesis of AD includes the interplay of environmental and genetic factors that cause derangements in the structure and function of the epidermal barrier and immune system [12,13] and even non-lesional skin seems to bear ultrastructural changes [14]. The main types of AD are extrinsic and intrinsic, differentiated by IgE levels, prevalence, clinical features, role of the filaggrin gene, and cytokine expression [15]. Extrinsic (or allergic) AD is burdened by high serum IgE levels, the presence of environment- or food-specific IgEs, and has a high prevalence. Meanwhile, intrinsic (or non-allergic) AD shows normal IgE values, the absence of specific IgE, and has a relatively low prevalence (20%) with female predominance and a late onset, milder clinical features, and higher interferon-γ expression [16]. Many studies focus on the complexity of cytokines and chemokines involved in the immune response of AD patients and the function of various cell types and epidermal barriers, which is perturbed in the extrinsic type of AD [4,13]. One of the crucial proteins in epidermal differentiation, filaggrin, facilitates the skin barrier formation [17,18] and a variety of *FLG* gene mutations were found in many patients with extrinsic AD. Cellular markers and adhesion molecules play a significant role in AD pathogenesis [19] via endothelial–leukocyte interactions, lymphocyte circulation, enhanced vascularization, and inflow of immunocompetent cells [20]. The two main types of AD are differentiated by higher expression of interleukin (IL) -4, IL-5, and IL-13, and the lower expression of interferon-γ in the extrinsic type [15]. IL-13 contributes to the pathogenesis of AD, although IL-4 is necessary for the Th2 cell polarization [21]. Defining the molecular cocktail typical for the AD type is crucial in the treatment decision. For example, inhibition of IL-4 and IL-13 is the main goal of dupilumab, an IgG monoclonal antibody used in the treatment of inadequately controlled AD, targeting the IL-4 receptor alpha subunit, thus inhibiting the IL-4 and IL-13 pathways [22].

Adhesion molecules, such as intercellular adhesion molecule–1 (ICAM-1), ICAM-3, E-selectin, and L-selectin, are highly expressed in lesions of patients with AD and play an essential role in the AD etiopathogenesis [23,24,25,26,27,28]. Intercellular adhesion molecule-1 (ICAM-1, CD54) is a 90 kDa member of the immunoglobulin (Ig) superfamily. It is critical for the transmigration of leukocytes from blood vessels to tissues, and is constitutively present on endothelial cells and increases in expression in response to pro-inflammatory cytokines. ICAM-1 acts as a leukocyte adhesion molecule and contributes to inflammatory responses by increasing endothelial cell activation [29]. Vascular cell adhesion molecule-1 (VCAM-1, CD 56), an adhesion molecule with Ig domains mainly expressed in endothelial cells, is strongly induced by inflammatory cytokines and plays a critical role in mediating leukocyte adhesion on endothelial cells, and activation of signaling pathways to facilitate leukocyte passage from blood to tissue. [30]. E-selectin (CD62E, endothelial–leukocyte adhesion molecule 1, leukocyte–endothelial cell adhesion molecule 2) is mainly expressed after inflammatory stimulation by activated endothelial cells, and is also expressed in bone marrow and skin. E-selectin binds to ligands expressed in polymorphonuclear and mononuclear leukocytes during inflammation [31].

These adhesion molecules stimulate selective migration of memory cutaneous leukocyte-associated antigen, as well as lymphocyte, monocyte, and granulocyte diapedesis in both skin and blood in AD. Studies have shown that adhesion molecules E-selectin, VCAM-1, and ICAM-1 are highly expressed on vascular endothelial cells in the skin of patients suffering from AD [20,25,26,28]. E-selectin is synthesized and expressed on the endothelium after stimulation by inflammatory cytokines. ICAM-1 and VCAM-1, as essential adhesion molecules in the migration of lymphocytes, correlate with the degree of dermal lymphocyte inflammation [32] and the latter is also significant in vasculogenesis in adults [33]. The determination of inflammation markers in the serum of patients with AD seems to be a valuable indicator of AD activity [23,24,25,26,27,34,35,36,37].

We aimed to determine the expression and topography of adhesion molecules E-selectin, ICAM-1, and VCAM-1 in the epidermis and dermis of atopic skin compared to healthy skin by comparing measurements by pathologists and a method of digital analysis. This correlation would present a valuable tool for disease assessment and follow-up of AD activity due to its simplicity and clinical value.

## 2. Materials and Methods

### 2.1. Biopsy Specimens (Patients)

**Figure 1:**
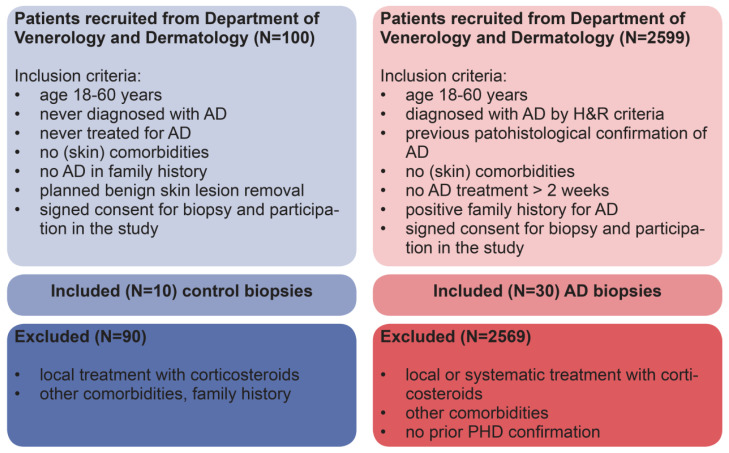
Flowchart of control patients (blue) and patients with atopic dermatitis (red) included in the study. N = number, AD = atopic dermatitis, H&R = Hanifin and Rajka atopic dermatitis clinical criteria [8].

This retrospective case–control study (level of evidence 2B) [38] included 30 patients aged 28–45 (mean age 32 years) (Table 1) who were diagnosed with AD using the clinical diagnostic criteria [8]. The patients had a diagnostic lesional skin biopsy performed earlier that confirmed the diagnosis and were not treated for at least two weeks prior to the study to eliminate any potential effect of corticosteroids or other immunosuppressive drugs. The specimens were archived at the Referral Centre for Contact Dermatitis, Department of Dermatology and Venereology, University Hospital Centre Zagreb. Skin biopsy specimens (punch biopsy technique provided full-thickness 4 mm skin specimens) were also obtained from the same site [39] from 10 healthy age-matched donors (Table 1) who had a regular appointment for benign skin lesion excision and were used as the control group. Informed consent for performing the biopsy, participating in this research, and data publication for this study was obtained from all included patients. Exclusion criteria for patients suffering from AD and controls are shown in Figure 1. All patient-related data were fully anonymized in the analysis within the study. The study protocol followed the EU guidelines and was approved by the ethical committee of the School of Medicine, University of Zagreb.

### 2.2. Immunohistochemistry

Biopsy specimens were formalin-fixed, paraffin-embedded, and sectioned with a microtome. The 4 μm thick skin sections from healthy participants and patients with AD were deparaffinized at 56 °C and immunohistochemically stained with E-selectin, ICAM-1, and VCAM-1 antibodies (Dako Animal Research Kit, Peroxidase; Dako, Code No. K3954, Dako, Glostrup, Denmark), following the manufacturer’s instructions and using the standard avidin–biotin immunoperoxidase staining method. Endogenous peroxidase activity was blocked with 0.3% hydrogen peroxide in Tris-buffered saline (TBS) for 15 min at room temperature (RT). The slides were then incubated with primary antibodies for E-selectin (clone 4.5a2 No M2063), ICAM-1 (clone 6.5b5, No M7063), and VCAM-1 (clone 1.4C3, No M7106), all diluted 1:80 with antibody dilution solution (No S2022) for 1 h at RT. The sections were incubated with a biotinylated secondary antibody (Dako LSAB Kit, No K678) for 30 min and streptavidin–peroxidase (No K678) for 30 min, also at RT. 3.3-diaminobenzidine tetrachloride (DAB) was used to visualize the staining. Between incubations, the slides were washed three times with TBS and counterstained with hematoxylin, washed in tap water, dehydrated through grades of alcohols to xylene, and covered with mounting media (Faramount media, DAKO) and a coverslip at RT.

### 2.3. Quantitative and Qualitative Staining Measurements

The expression of adhesion molecules in the epidermis and dermis of control and patients with AD was analyzed by evaluating the incidence and intensity of positively stained areas in the tissue sections. This descriptive analysis was used to determine the topographical scatter of molecular expression by classifying the staining intensity as negative, weak, moderate, or strong and was performed by two pathologists.

To quantify changes in the expression of adhesion molecules, the number of epidermal ICAM-1, VCAM-1, and E-selectin positive cells was measured digitally [40]. The number of positively stained cells was expressed as a percentage of positive epidermal or endothelial cells in all epidermal or endothelial cells per microscopical field, respectively.

Positive ICAM-1, VCAM-1, and E-selectin epidermal surface area in control and AD-affected skin, as well as endothelial surface areas in the dermal blood vessel wall, were measured using the method of digital image analysis described previously [41]. The data were expressed as the percentage of E-selectin-, ICAM-1-, and VCAM-1-positive epidermal/endothelial surface area, respectively, in the whole analyzed epidermal or endothelial surface area in a microscopical field, depending on where the measurement was performed. 

All measurements and staining analysis were performed on digital images of skin tissues (Nikon Eclipse E600 light microscope and Nikon DXM1200 digital camera (Nikon, Kingston-upon-Thames, UK)) using the Imaging Software Lucia G 4.80 (Laboratory Imaging Ltd., Prague, Czech Republic). These measurements were performed on six fields per section at 1000× magnification.

### 2.4. Biopsy Specimens (Patients)

The sample size was determined in a pilot study, performed prior to the main data collection, and supported by data obtained from the literature. The data were statistically analyzed using the Statistica 6.0 software package (StatSoft, Tulsa, OK, USA). Descriptive analysis was performed for all data. Statistical significance of the difference in the percentage and the overall number of the immunostained tissue area was tested using the Student’s *t*-test. Tables and graphs show the mean values and standard errors of the mean. The statistical significance level was *p* < 0.05.

## 3. Results

### 3.1. E-Selectin

Immunohistochemical analysis of skin from healthy controls and lesional skin from patients with AD showed expression of E-selectin in both the epidermis and dermis.

**Figure 2:**
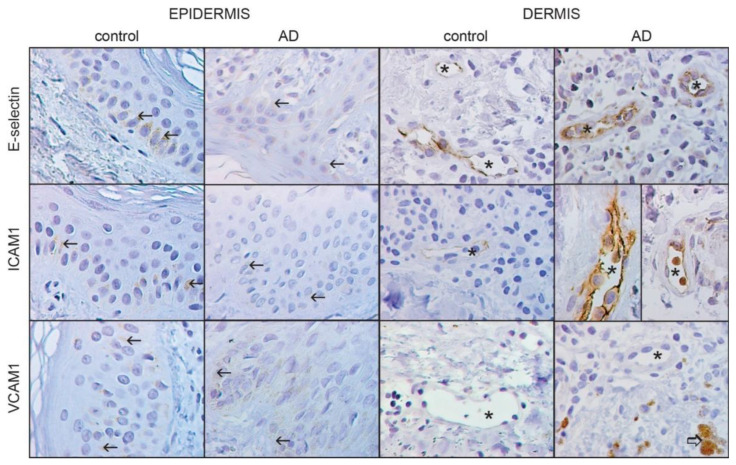
Representative images of immunohistochemical staining in the epidermis and dermis in controls and skin affected by atopic dermatitis. The staining of E-selectin, ICAM1, and VCAM1 showed differences in expression. Arrows (→) mark the DAB-positive signal in the cell cytoplasm, asterisks (*) show the vascular lumen, and the thick arrow (⇨) shows macrophages. DAB, hematoxylin counterstain, 1000× magnification.

In the epidermis of healthy control skin, E-selectin was expressed in most cells of the basal layer and some cells of the suprabasal layer. The strongest staining was observed in the cytoplasm around the nucleus. In the epidermis of AD-affected skin, E-selectin expression was also found in the cells of the basal and suprabasal layer. However, the staining was weaker than in the control group (Figure 2).

**Figure 3:**
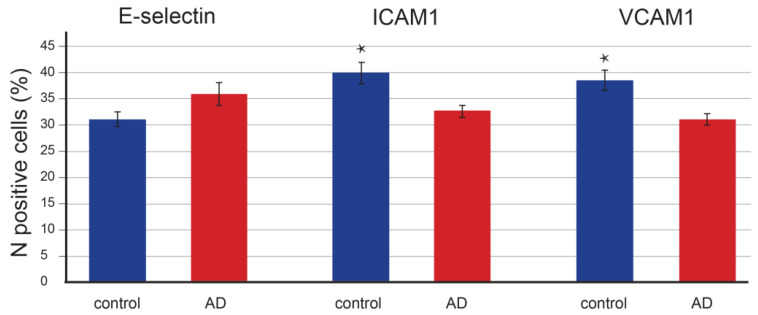
The number of DAB-positive epidermal cells in controls and skin affected by atopic dermatitis. The number is expressed as the percentage of immunostained epidermal cells in the overall epidermal cell number. The number did not significantly change for the E-selectin-positive signal; it decreased by 1.2× in AD patients compared to controls (*p* = 0.005) for ICAM-1-positive epidermal cells and decreased by 1.3× in AD patients (*p* < 0.001) for VCAM-1-positive cells. Data are presented as mean ± SEM. Student’s *t*-test, two-tailed; * *p* < 0.001.

**Figure 4:**
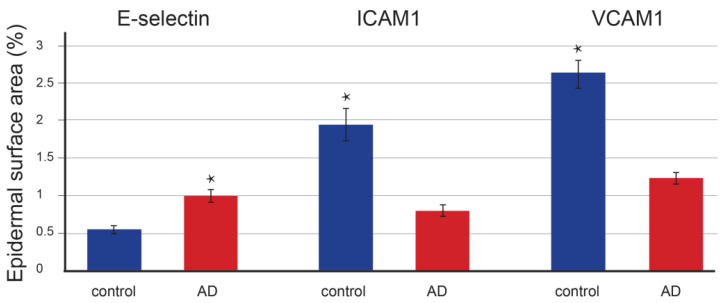
The epidermal surface area in controls and skin affected by atopic dermatitis. The area surface is expressed as a percentage of immunostained epidermal area in the overall epidermal area. The E-selectin-positive epidermal area was significantly increased (*p* < 0.001) in AD patients, while ICAM1- and VCAM1-positive epidermal areas were decreased in AD patients compared to controls: ICAM1 2.5× (*p* < 0.001) and VCAM1 2× (*p* < 0.001). Data are presented as mean ± SEM. Student’s *t*-test, two-tailed; * *p* < 0.001.

**Figure 5:**
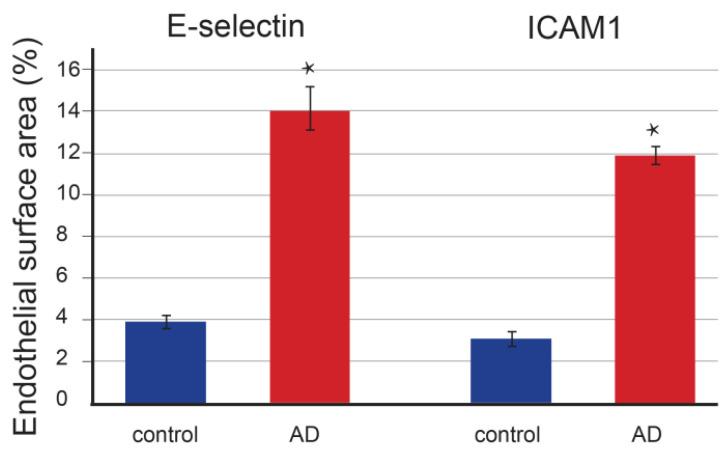
The endothelial surface area in controls and skin affected by atopic dermatitis. The area surface is expressed as a percentage of the immunostained endothelial vessel wall area in the overall endothelial area. The E-selectin-positive endothelial area was significantly larger (3.5×, *p* < 0.001) in AD skin and the ICAM1-positive area was almost 4× larger (*p* < 0.001) in AD skin. Data are presented as mean ± SEM. Student’s *t*-test, two-tailed; * *p* < 0.001.

Quantitative staining analysis revealed that the number of E-selectin-positive epidermal cells in control (*p* = 0.097) and AD-affected (*p* = 0.091) skin did not significantly differ (Figure 3). In contrast, the positive epidermal area was significantly increased (*p* < 0.001) in patients with AD (Figure 4) compared to controls (*p* = 0.064). In the dermis, E-selectin expression was found in the endothelial cells of blood vessels as a moderately stained reaction. However, the staining was much stronger in AD-affected skin than in control skin, with a strong granular signal in macrophages (Figure 2). The quantification also revealed significant changes in the endothelial expression in the form of an enlarged positive endothelial area in the vessel wall, which was 3.5 times higher (*p* < 0.001) in AD-affected skin compared to control skin (*p* = 0.073) (Figure 5).

### 3.2. ICAM-1

In the epidermis of control skin, ICAM-1 was expressed in most cells of the basal layer and some cells of the suprabasal layer. The strongest staining was found around the nucleus. In the skin of patients with AD, ICAM-1 expression showed a similar topographical pattern, although with weaker intensity (Figure 2). The quantitative analysis showed a significant decrease in epidermal expression in patients with AD. The number of positive cells decreased 1.2 times (*p* = 0.005 vs. *p* = 0.055, Figure 3), and the positive epidermal area decreased 2.5 times compared to the control group (*p* < 0.001 vs. *p* = 0.67, Figure 4).

In the dermis of control skin, ICAM-1 was weakly expressed only in vascular endothelial cells. On the contrary, in AD-affected skin, the staining was very strong in almost all endothelial cells (Figure 2).

The quantitative staining analysis also revealed a significant increase in endothelial expression of ICAM-1 in the vessel wall in the form of an enlarged positive endothelial area, which was 3.8 times larger (*p* < 0.001, Figure 5) than that in control skin (*p* = 0.077).

### 3.3. VCAM-1

VCAM-1 was expressed almost in all layers of the epidermis, with the strongest staining observed in the suprabasal layer cells. However, the expression was significantly weaker in AD-affected skin than in control skin (Figure 2).

Quantifying the immunostained area revealed a significant decrease in epidermal expression in AD-affected skin. The number of positive cells decreased 1.3 times (*p* < 0.001 vs. *p* = 0.121, Figure 3) and the positive area decreased by half compared to the control (*p* < 0.001 vs. *p* = 0.095, Figure 4).

In the dermis of control and AD-affected skin, VCAM-1 expression was found only in the macrophage-like cells of AD-affected skin (Figure 2), and therefore, no quantitative analysis of endothelial expression could be performed.

## 4. Discussion

This study showed a detailed spatial arrangement of E-selectin, ICAM-1, and VCAM-1 expression and the difference in the molecular scatter in healthy and AD-affected skin. We found E-selectin to have a moderate expression in basal and suprabasal epidermal layers of controls and weak expression in the case of patients with AD. In the dermis, AD-affected skin showed an intense E-selectin signal in the vascular endothelium and macrophages with a weak signal in the endothelium of controls. ICAM-1 was weakly expressed in the basal and suprabasal epidermal layers of healthy and AD-affected skin. The vascular endothelium in the dermis showed a strong signal for ICAM-1 in patients with AD and a weak one in controls. VCAM-1 expression was found in all epidermal layers of controls but not the dermis. In patients with AD, VCAM-1 expression was scarce in the epidermal basal and suprabasal layers, strong in dermal macrophages, and non-existent in the vascular endothelium.

In this study of adhesion molecules in AD, we confirmed their presence and proved their importance as immunological factors involved in the pathogenesis of AD. There has been evidence of expression of adhesion molecules E-selectin, VCAM-1, and ICAM-1 in the skin of patients with AD [23,24,25,26]. These molecules are important in the allergic inflammation in AD because they stimulate the migration of cutaneous memory T-lymphocytes with cutaneous leukocyte-associated antigen, as well as lymphocyte, monocyte, and granulocyte diapedesis in skin and blood [20,28,32]. Soluble E-selectin and soluble ICAM-1 are a marker of the activity of AD in children [28]. Our results also agree with those of an earlier study [42] showing that adhesion molecules play a crucial role in allergic inflammation because they induce selective migration of T-lymphocytes expressing CLA, thereby enabling diapedesis of cells such as monocytes and granulocytes [37]. Increased levels of endothelial leukocyte adhesion molecules ICAM-1 and VCAM-1 have been found in the tissue and serum samples obtained from patients with AD [23,24,25,26,27,28].

Although studies in patients suffering from AD showed evidence supporting a contributing role of ICAM-1 and VCAM-1 in AD by proof of higher dermal vascular expressions of these two molecules, there has been evidence of adhesion molecule ICAM-1 in the endothelium, in cells surrounding blood vessels, and in the (supra)basal layer of the epidermis [19,20,24,28]. ICAM-1 is a critical adhesion molecule in lymphocyte migration into the dermis, which is in correlation with the stage of dermal lymphocyte inflammation, and it is considered the most crucial adhesion molecule in the etiopathogenesis of AD. ICAM-1 expression was identified in the epidermis’s basal and suprabasal layers and the walls of blood vessels in the dermis. These findings are consistent with the findings of Wüthrich et al., who proved the expression of ICAM-1 on keratinocytes, fibroblasts, lymphocytes, and perivascular cells [24].

Some inconsistencies were found when comparing our results with the work of other researchers saying that VCAM-1 is an adhesion molecule expressed in endothelial cells and perivascular dermis-infiltrating cells, presenting an essential parameter for estimating inflammation activity in AD [23,24]. Our results demonstrated that VCAM-1, much more than ICAM-1, contributes to developing skin inflammation in AD.

This study’s data contribute to the understanding of the etiopathogenesis of AD. Our study led us to conclude that E-selectin might be the most sensitive parameter for estimation of the clinical course of AD. This study demonstrated the presence of changes in organization and topographical scatter of adhesion molecules in the skin that are specific for AD. These findings further support the hypothesis that adhesion molecules play a vital role in the pathogenesis of AD. 

In order to maximally avoid bias caused by different scoring categories, non-standardized approaches, and variability of visual analysis, a digital image analysis method was used, which was described previously as an optimal method when the dataset is not too large and the analyzed tissue is histologically homogenous [40,41].

Further studies might include a comparison of adhesion molecule expression in lesional and non-lesional AD-affected skin [14] on the T-cell receptor repertoire. 

Despite many different studies on the etiopathogenesis of atopic dermatitis, this issue remains an unsolved puzzle for further investigations of immunological factors responsible for inflammation in this disease.

In conclusion, AD-affected skin expressed E-selectin, ICAM-1, and VCAM-1 in a disease-specific way. E-selectin may be the most sensitive parameter for estimation of the clinical course of AD. The topographical scatter and specific changes in the organization of adhesion molecules in the skin that are specific for AD further support the hypothesis that adhesion molecules play a vital role in the pathogenesis of AD.

## Figures and Tables

**Table 1:** Demographic data of patients included in the study. N = number.

	Patients with Atopic Dermatitis	Controls
N	30	10
N females	21	5
N males	9	5
age	28–45	24–32
mean age	32	28

## Data Availability

Not applicable.
